# Influence of triangular obstacles on droplet breakup dynamics in microfluidic systems

**DOI:** 10.1038/s41598-024-63922-y

**Published:** 2024-06-10

**Authors:** Azadeh Tazikeh Lemeski, Seyyed Masoud Seyyedi, Mehdi Hashemi-Tilehnoee, Azadeh Sadat Naeimi

**Affiliations:** 1 Department of Mechanical Engineering, Aliabad Katoul Branch, Islamic Azad University, Aliabad Katoul, Iran; 2 Centre for Cooperative Research on Alternative Energies (CIC energiGUNE), Basque Research and Technology Alliance (BRTA), Alava Technology Park, Albert Einstein 48, 01510 Vitoria-Gasteiz, Spain; 3 Department of Physics, Aliabad Katoul Branch, Islamic Azad University, Aliabad Katoul, Iran

**Keywords:** Multiphase, T-junction microchannel, A triangle obstacle, Droplet breakup, Engineering, Biomedical engineering, Mechanical engineering

## Abstract

Microfluidic devices with complex geometries and obstacles have attracted considerable interest in biomedical engineering and chemical analysis. Understanding droplet breakup behavior within these systems is crucial for optimizing their design and performance. This study investigates the influence of triangular obstacles on droplet breakup processes in microchannels. Two distinct types of triangular obstructions, positioned at the bifurcation (case I) and aligned with the flow (case II), are analyzed to evaluate their impact on droplet behavior. The investigation considers various parameters, including the Capillary number (Ca), non-dimensional droplet length (L*), non-dimensional height (A*), and non-dimensional base length (B*) of the triangle. Utilizing numerical simulations with COMSOL software, the study reveals that the presence of triangular obstacles significantly alters droplet breakup dynamics. Importantly, the shape and location of the obstacle emerge as key factors governing breakup characteristics. Results indicate faster breakup of the initial droplet when the obstacle is positioned in the center of the microchannel for case I. For case II, the study aims to identify conditions under which droplets either break up into unequal-sized entities or remain intact, depending on various flow conditions. The findings identify five distinct regimes: no breakup, breakup without a tunnel, breakup with a tunnel, droplet fragmentation into unequal-sized parts, and sorting. These regimes depend on the presence or absence of triangular obstacles and the specific flow conditions. This investigation enhances our understanding of droplet behavior within intricate microfluidic systems and provides valuable insights for optimizing the design and functionality of droplet manipulation and separation devices. Notably, the results emphasize the significant role played by triangular obstacles in droplet breakup dynamics, with the obstacle’s shape and position being critical determinants of breakup characteristics.

## Introduction

In recent times, there has been an increasing interest in the development of microfluidic devices in both academic and industrial fields^1^. This is due to the unique characteristics of microfluidic devices, such as their low cost, high efficiency, controllability, and safety. Microfluidic devices are capable of processing small amounts of fluid ranging from a nanoliter to a milliliter. They are composed of microfluidic channels, inlet/outlet ports, and other relevant micro-features that enable them to function.

These benefits have facilitated the utilization of microfluidic devices in a wide range of industrial applications^2^. These applications include protein crystallization^3–5^, emulsification^6–9^, lab-on-a-chip systems^10,11^ and drug discovery^12^. There are different processes, involving the coalescence^13^, formation^14–20^, breakup in symmetric^21–24^ and asymmetric^25–32^ geometries.

A wide range of experimental and numerical studies are employed to investigate fluid flow phenomena in diverse fields, including two phase flow^33,34^, porous media, unsteady flow^35,36^, aneurysm^37,38^, Supersonic flow^39,40^, and the manipulation of droplets in microfluidic devices^14–23^. According to Link et al.^27^, two methods for passively breaking larger droplets into unequal-sized droplets are using an obstacle inside a straight channel and a T-junction with unequal length branches. However, the main disadvantage of using an obstacle inside a straight channel is that after generating large and small droplets, the produced small and large droplets move together along the channel, and a different process is required to separate them. Choi et al.^28^ introduced a novel technique for generating droplets of unequal sizes using multi-layer microfluidic chips. In this method, droplets pass through a constriction formed by a pneumatically actuated valve, which breaks them into smaller droplets. The volume ratio of the droplets can be controlled by adjusting the gas pressure of the control valve channel, as even a small change in pressure can alter the volume ratio. Ting et al.^29^ proposed an effective method for controlling droplets in a microchannel that involves a heater in one of the branches of a symmetric T-junction. In the heated branch, the size of the droplet is smaller than that of the droplet in the other branch. However, one of the limitations of this method is that the temperature must be kept below 55 °C. Bedram and Moosavi^30^ conducted a numerical investigation of the breakup of non-uniform droplets in an asymmetric T-junction microchannel with an inlet channel and two different-sized outlet channels. Their results showed that smaller droplets can be produced by increasing Ca. They also found that the breakup time and pressure drop for this system are smaller than those for a symmetric system with different-length outlet channels. Dupin et al.^14^ proposed a method for generating droplets of a specific size. Their practical model is highly efficient and supports a wide range of parameters, including surface wetting, surface tension, liquid–liquid wetting, viscosity ratio, and inlet velocity. Wu et al*.*^41^ and Liu and Zhang^15,16^ have also conducted research on droplet formation in microchannels. However, their methods do not involve the breakup of droplets into multiple parts with equal or unequal sizes. This limitation restricts their applications compared to the method proposed by Dupin et al.^14^. Another approach was described by Aboutalebi et al.^32^, who numerically investigated the splitting of magnetic microdroplets in symmetric T-junctions under an asymmetrically applied magnetic field. They established a correlation between the splitting and non-splitting zones of microdroplet flow in micro T-junctions and presented the effect of magnetic force on ferrofluid microdroplets.

Another highly efficient technique for generating droplets involves the strategic placement of an obstacle within the trajectory of the microchannel. By introducing an obtrusion in the path of the microchannel, the fluid flow is disrupted, resulting in the formation of distinct droplets. Changkwon et al.^42^ investigated the effect of droplet size and Ca on droplet dynamics for both Newtonian and viscoelastic fluids. The study revealed that a Newtonian droplet immersed in a viscoelastic medium breaks up into two smaller droplets while passing through a cylinder obstruction with an increasing Deborah number. The authors reported that the normal stress difference plays a crucial role in droplet extension and breakup. Additionally, Changkwonthe et al.^43^ explored droplet dynamics passing through obstructions in a confined microchannel using both numerical and experimental methods. They investigated the effects of obstruction shape (cylinder and square), droplet size, and Ca on droplet dynamics. The main disadvantage of this strategy^42,43^ is that a different process is needed to separate small and large droplets in the path of microchannel.

Current research on the droplet breakup in microfluidic devices primarily focuses on methods like obstacles within channels or T-junction geometry variations^27^. While these approaches offer some degree of control, they often present challenges in separating the generated droplets of different sizes^27^. Additionally, methods involving microfluidic valves or temperature control introduce complexities and limitations^28,29^. Here, this gap is addressed by exploring a novel and potentially more versatile approach for achieving droplet breakup: utilizing triangular obstacles strategically placed within T-junction microchannels. The influence of obstacle shape on droplet breakup dynamics has been investigated previously^42,43^. However, the impact of triangular obstacles on this process remains largely uncharacterized. This lack of knowledge presents a significant opportunity. Therefore, the authors conducted a systematic study to investigate the effect of a triangle obstacle on droplet breakup processes. Several non-dimensional parameters including Capillary number, non-dimensional droplet length, non-dimensional height of the triangle, and non-dimensional base length of the triangle are investigated in details.

## Numerical simulation

### Geometry of the microchannel

**Figure 1:**
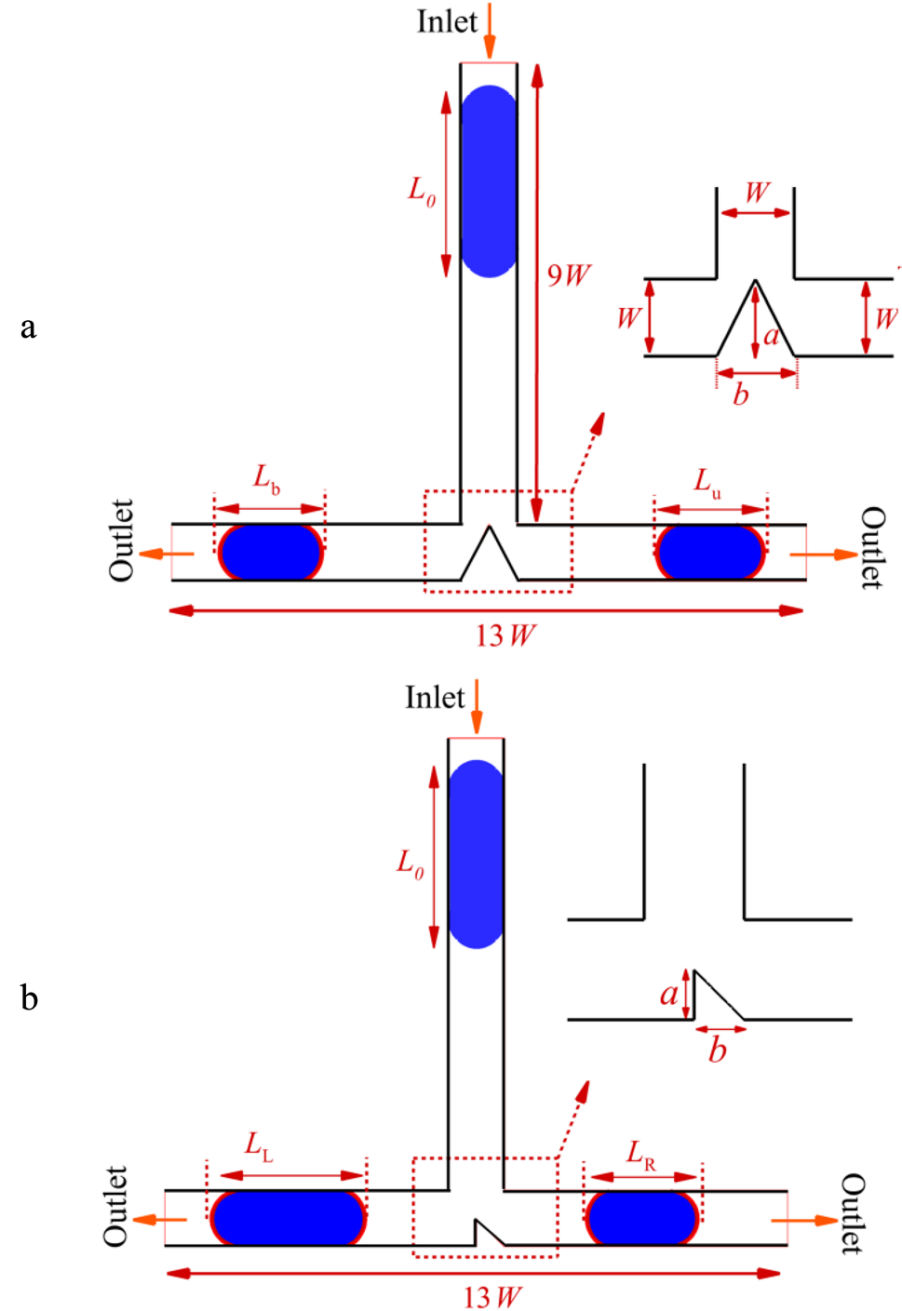
Schematic of a two-dimensional T-junction microchannel.

Figure 1 illustrates a schematic diagram depicting the passage of a droplet through a T-junction microchannel with an obstacle. The microchannel consists of a single inlet and two outlets, with a triangular obstacle positioned at the T junction. Two types of triangle obstructions are considered to investigate their effects: (a) a triangle located at the bifurcation (Case I), and (b) an aligned triangle (Case II). The triangle has a base length of *b* and a height of *a*. The continuous phase, labeled as 1, represents the fluid medium through which the droplet passes. The dispersed phase, labeled as 2, corresponds to the droplet phase suspended within the continuous phase. The densities of the continuous and dispersed phases are represented by $\rho_{1}$ and $\rho_{2}$, respectively. Both the inlet channel and outlet have a width equal to *W* $\left( { = 100} \right)$ μm.The initial length of the droplet is denoted as *L*_0_. The coordinates *x* and *y* represent the horizontal and vertical components of the position, respectively.

The physical properties of fluids are presented in Table 1.

**Table 1:** Physical properties of droplet and the surrounding fluid^25^.

	Density (kg/m^3^)	Viscosity (mPa s)	Surface tension (N/m)
Disperse fluid	1000	1	0.0033
Continuous fluid	930	10	

The boundary conditions for the simulation are as follows: a uniform velocity profile is set at the inlet, and an outflow boundary condition is considered at the outlet, which assumes a fully developed flow field at the end of the main channel. This deforms the shape of droplets that reach the outlet, as the Laplace pressure jump across an interface is forced to zero. Additionally, it makes a curved interface become perpendicular to the outlet. However, this does not significantly affect droplets upstream. The walls are subject to a non-slip boundary condition.

### Governing equations and numerical method

The mass conservation and momentum conservation equations for incompressible fluid can be expressed as follows when simulating the deformation of a droplet in a microchannel^44^:

Mass Conservation Equation:

1 $$\nabla { } \cdot {\varvec{u}} = 0.$$

Momentum Conservation Equation:

2 $$\rho \frac{{\partial {\varvec{u}}}}{{\partial {\text{t}}}} + \rho \left( {{\varvec{u}} \cdot \nabla } \right){\varvec{u}} = - \nabla p + \nabla \cdot \left[ {\mu \left( {\nabla { }{\varvec{u}} + \left( {\nabla {\varvec{u}}} \right)^{T} } \right)} \right] + {\varvec{F}}_{\sigma } { }.$$

where the variables $p$, $\rho$, ${\varvec{u}}$ and ${\varvec{F}}_{\sigma }$ represent the pressure, density, velocity, and interfacial force of two fluids that do not mix. The surface tension force, denoted as ${\varvec{F}}_{\sigma }$ in Eq. (2), is diffused within a narrow region surrounding the interface of the two phases. Its expression can be described as follows:

3 $${\varvec{F}}_{\sigma } = \sigma \kappa \delta {\varvec{n}}.$$

here $\sigma$ represents the surface tension coefficient, $\kappa$ stands for the curvature of the interface, and ${\varvec{n}}$ represents the normal direction with respect to the droplet surface. Additionally, $\delta$ is a Dirac Delta function.

To capture the interface of the droplet in this research, the Level-Set method is utilized. Two-Phase level-set method is a numerical technique that uses a fixed mesh to represent moving interfaces or boundaries. It is particularly useful for problems where the computational domain can be divided into two regions separated by an interface. The method introduces an additional scalar field, known as the level set function ($\Phi$), which smoothly varies between − 1 and + 1 to define the interface between two immiscible fluids. The governing equations for this method are a type of convection–diffusion equation, which can be numerically challenging to solve due to the significant advective term and abrupt transition in the field.

The level-set function, represented as $\Phi$, is calculated using its governing equation:

4 $$\frac{\partial \Phi }{{\partial {\text{t}}}} + \nabla \cdot \left( {{\varvec{u}} \Phi } \right) = \lambda \nabla \cdot \left( {\epsilon \nabla \Phi - \Phi \left( {1 - \Phi } \right)\frac{\nabla \Phi }{{\left| {\nabla \Phi } \right|}}} \right).$$

where the symbols λ and $\epsilon$ correspond reinitialization parameter, and interface thickness parameter, respectively.

The density and viscosity are defined as $\rho \left( \Phi \right) = \left( {1 - \Phi } \right)\rho_{1} + \Phi \rho_{2}$ and $\mu \left( \Phi \right) = \left( {1 - \Phi } \right)\mu_{1} + \Phi \mu_{2}$, respectively.

Computational fluid dynamics (CFD) is a widely adopted technique in various industries for modeling real-world engineering problems. One well-known software used for simulating the movement of droplets under an electric field is COMSOL Multi-physics. In this approach, the fluid dynamics module of COMSOL Multi-physics employs the laminar Two-Phase level-set method to simulate the motion of droplets within a microchannel.

The following non-dimensional numbers are introduced that describe the problem;

$${\text{Ca}} = \frac{{{ }\mu_{1} { }u_{1} }}{\sigma }. {\text{L}}^{*} = \frac{{L_{0} }}{W}. {\text{A}}^{*} = \frac{a}{W} \,and \,{\text{B}}^{*} = \frac{b}{W}$$

where Ca, L*, A* and B* represents the Capillary number, non-dimensional droplet length, non-dimensional height of the triangle, and non-dimensional base length of the triangle, respectively.

## Results and discussion

To ensure the accuracy of the obtained results, a grid sensitivity test is carried out in advance of the execution of the numerical simulations. The primary aim of this examination is to ascertain the most optimal quantity of mesh elements to be employed, as it is crucial to strike a balance that would not compromise the precision of the results. An in-depth analysis of the data presented in Table 2 showcases the variations in normalized neck thickness that are observed for different quantities of meshing elements at a specific time point, denoted as t* = 10.5. It becomes apparent upon careful examination of the table that augmenting the total number of elements from 55,553 to 74,322 did not yield any discernible modifications in the normalized neck thickness. Hence, it can be confidently stated that the grid system that encompassed a total of 55,553 mesh elements is deemed to be the most suitable and appropriate choice for the T-junction simulation.

**Table 2:** Grid independency test: the variations in normalized neck thickness ($\delta /W$) for different quantities of meshing elements at a specific time point (t* = 10.5) for Ca = 0.013 and t* = 10.5.

Number of elements	$\delta /W$	% Difference
22,754	0.341	6.3
38,486	0.318	3.2
55,553	0.308	0.5
74,322	0.306	–

**Figure 2:**
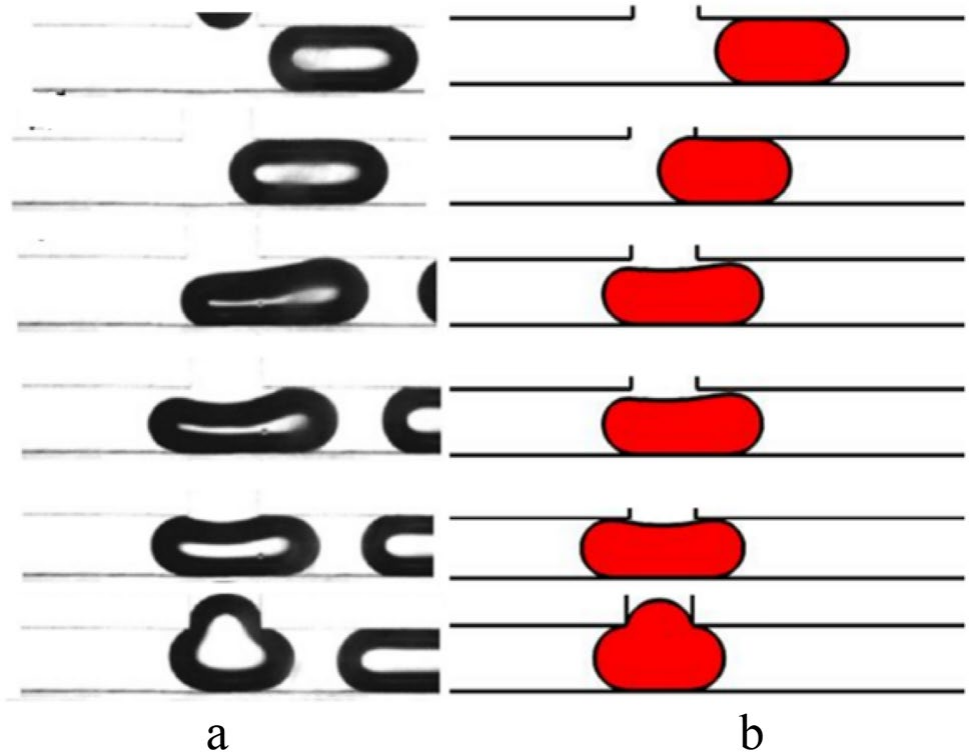
A comparison of the simulation results for the non-breakup regime with Ca = 0.0035 and L* = 3.2 is shown in: (**a**) the experimental result by Lu et al.^22^, and (**b**) current study.

**Figure 3:**
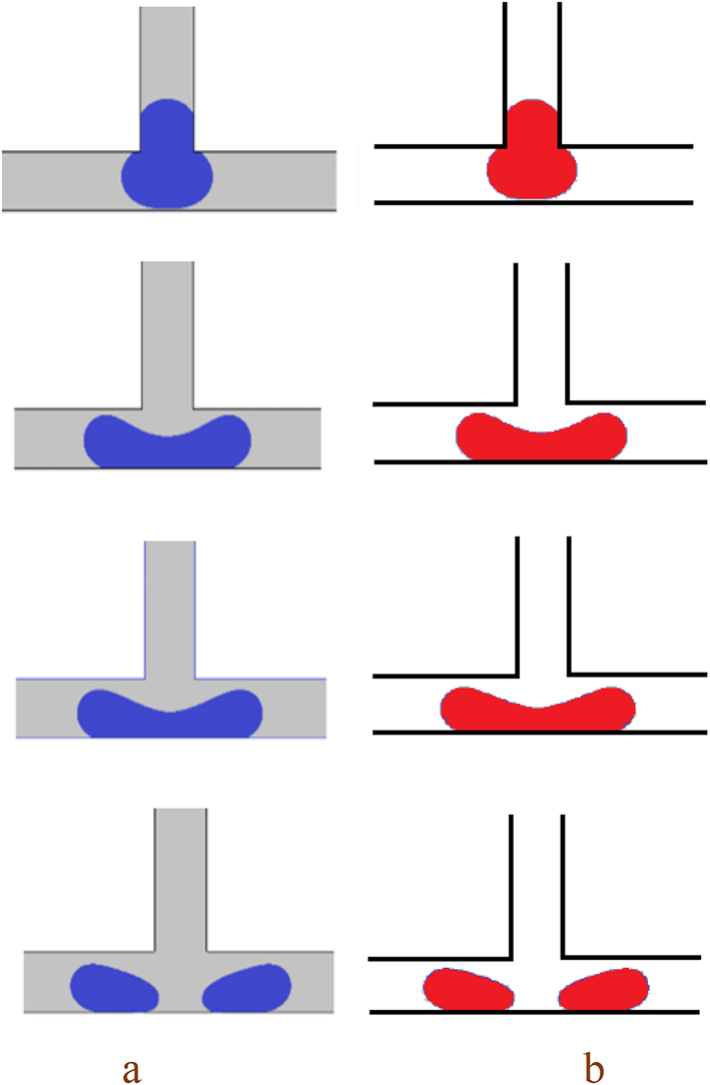
A comparison of the simulation results for the breakup with tunnel regime is shown in: (**a**) the numerical result by Deka et al.^45^, and (**b**) current study.

The droplet motion in a symmetric T-junction microchannel has been categorized into three different regimes by previous studies^10–15^. These regimes depend on the non-dimensional droplet length and the Capillary number. They are: non-breakup, breakup without tunnel, and breakup with tunnel. To verify the accuracy of the simulation, the current results are compared with the experimental result of Lu et al.^22^ for non-breakup and numerical result of Deka et al*.*^45^ for breakup with tunnel and are presented in Figs. 2 and 3, respectively. Figure 2 shows these results for the non-breakup regime. The surface tension force, the shear force, and the pressure difference force affect the droplet motion. The surface tension force is a powerful resistance force that stops the mother droplet from breaking into two daughter droplets in the non-breakup regime. The shear force and the pressure difference force, which are driving forces, cannot overcome the surface tension force. As a result, the mother droplet randomly goes to either the left or the right outlet arm. In the absence of external forces, the interplay between the droplet's surface tension and the channel's wettability determines its final direction. Also, it must take into account that many authors^46,47^ reported in their numerical results that droplet randomly flows one of the branches. Furthermore, Fig. 3 likely showcases the contrasting scenario where the shear forces overcome the surface tension, causing the mother droplet to break up and travel through the tunnel as two equal daughter droplets.

**Figure 4:**
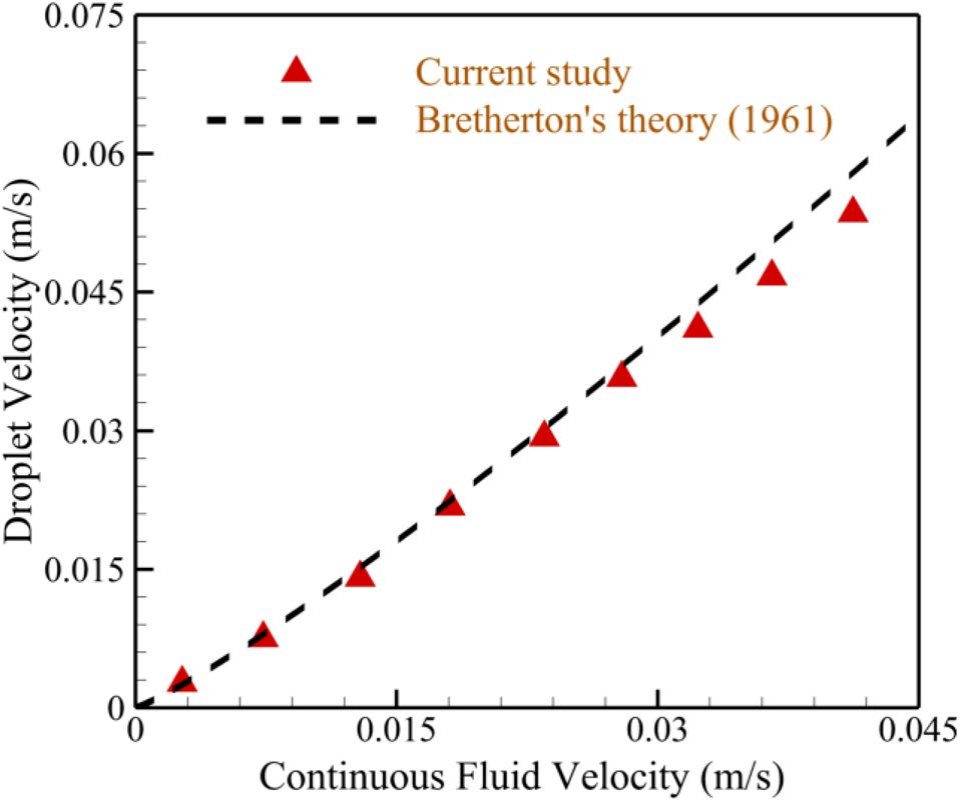
Verification of current study with Bretherton^48^ in the field of droplet velocity as a function of mean velocity of the carrier fluid.

To quantitatively validate our findings, a detailed comparison is made in Fig. 4 between the outcomes of the present research and the analytical relationship proposed by Bretherton^48^. It is assumed that the Reynolds number is small and there is no interaction between the dispersed phase and the tube wall. Bretherton introduced an analytical correlation for the velocity of the dispersed phase within a slender tube, taking into account the average velocity of the fluid flow, the surface tension, and the viscosity of the continuous phase. This correlation is expressed as:

5 $$U = \overline{U}\left( {1 + 1.29\left( {\frac{{\mu_{c} u_{c} }}{\gamma }} \right)^{\frac{2}{3}} } \right).$$

The results obtained from the current study exhibit a remarkable agreement with Eq. (5), as clearly illustrated in Fig. 4.

It is deducible from the data presented in Figs. 2, 3, and 4 that a high level of concurrence exists between the outcomes of the simulation carried out in this study and the data reported in earlier publications. Hence, it is equally plausible to assert that the model utilized in this research is a suitable approach for examining the dynamics of droplets within a T-junction microchannel with triangular obstacles.

**Figure 5:**
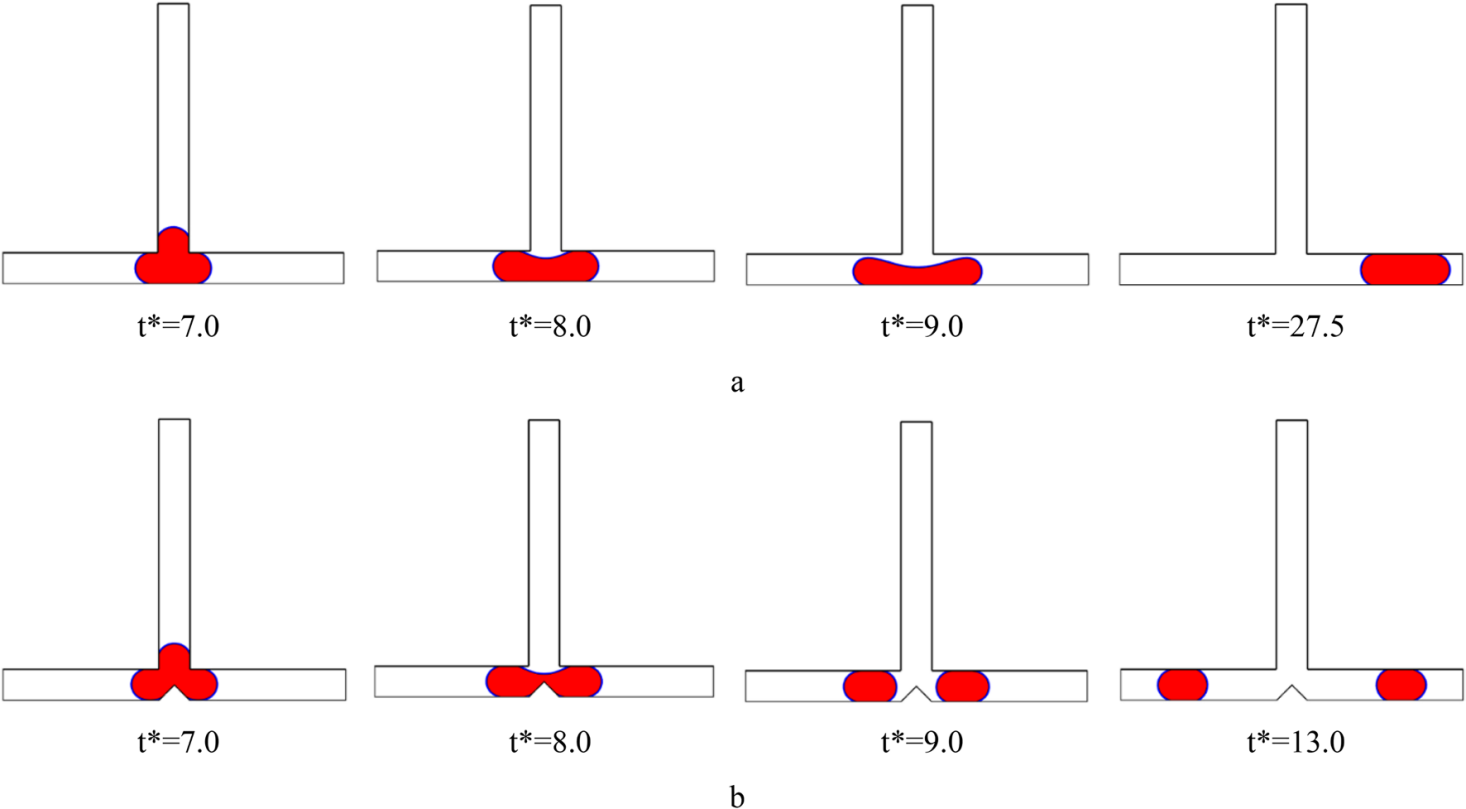
Snapshots of the droplet’s movement in the symmetric T-junction microchannel at L* = 3.0 for Ca = 0.013: (**a**) without an obstacle and (**b**) with triangle at the branching point (case I: B* = *b*/*W* = 1.0 and A* = *a*/*W* = 0.5).

**Figure 6:**
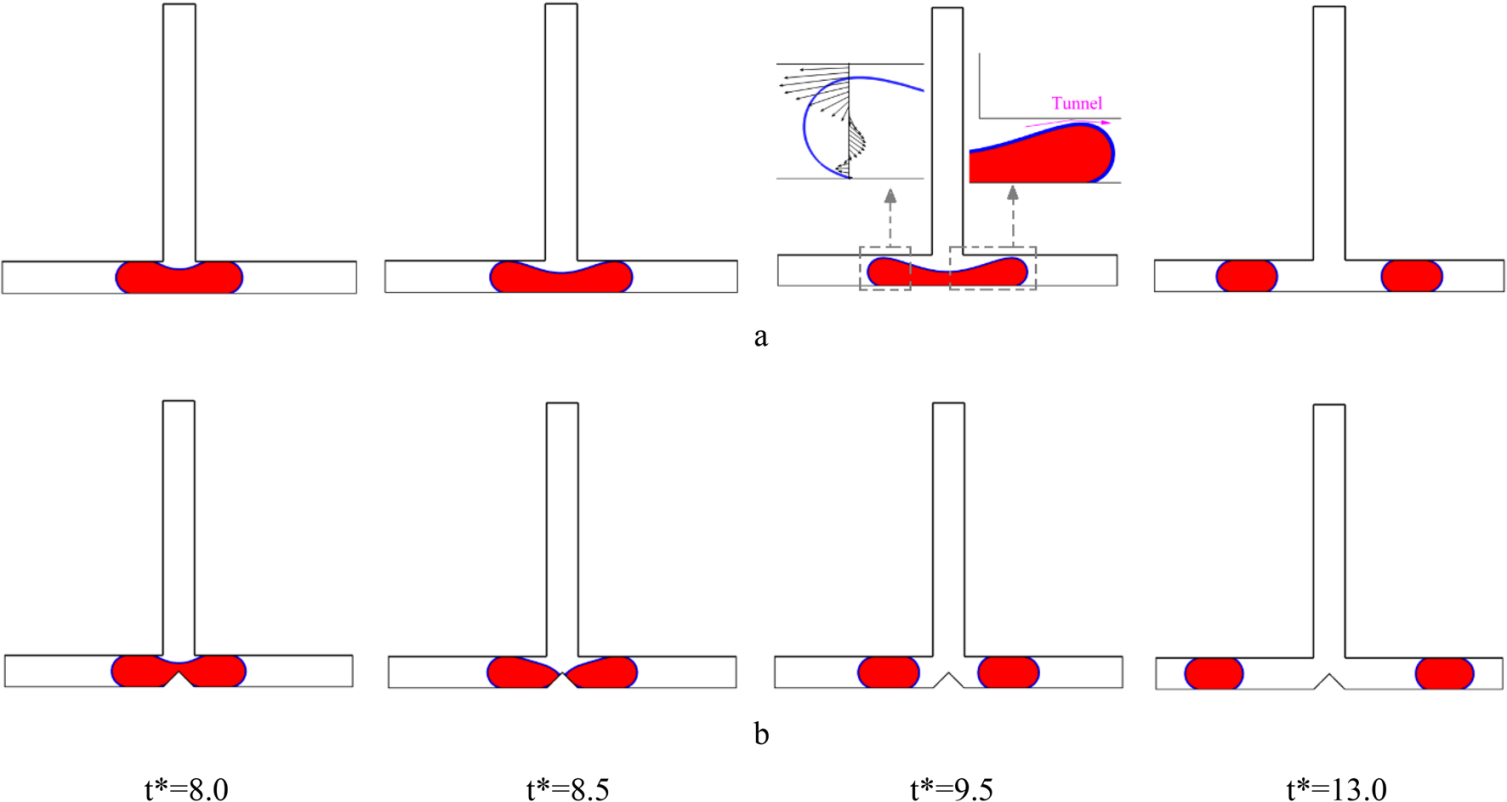
Snapshots of the droplet’s movement in the symmetric T-junction microchannel at L* = 3.5 for Ca = 0.013: (**a**) without an obstacle and (**b**) with triangle at the branching point (case I: B* = *b*/*W* = 1.0 and A* = *a*/*W* = 0.5).

**Figure 7:**
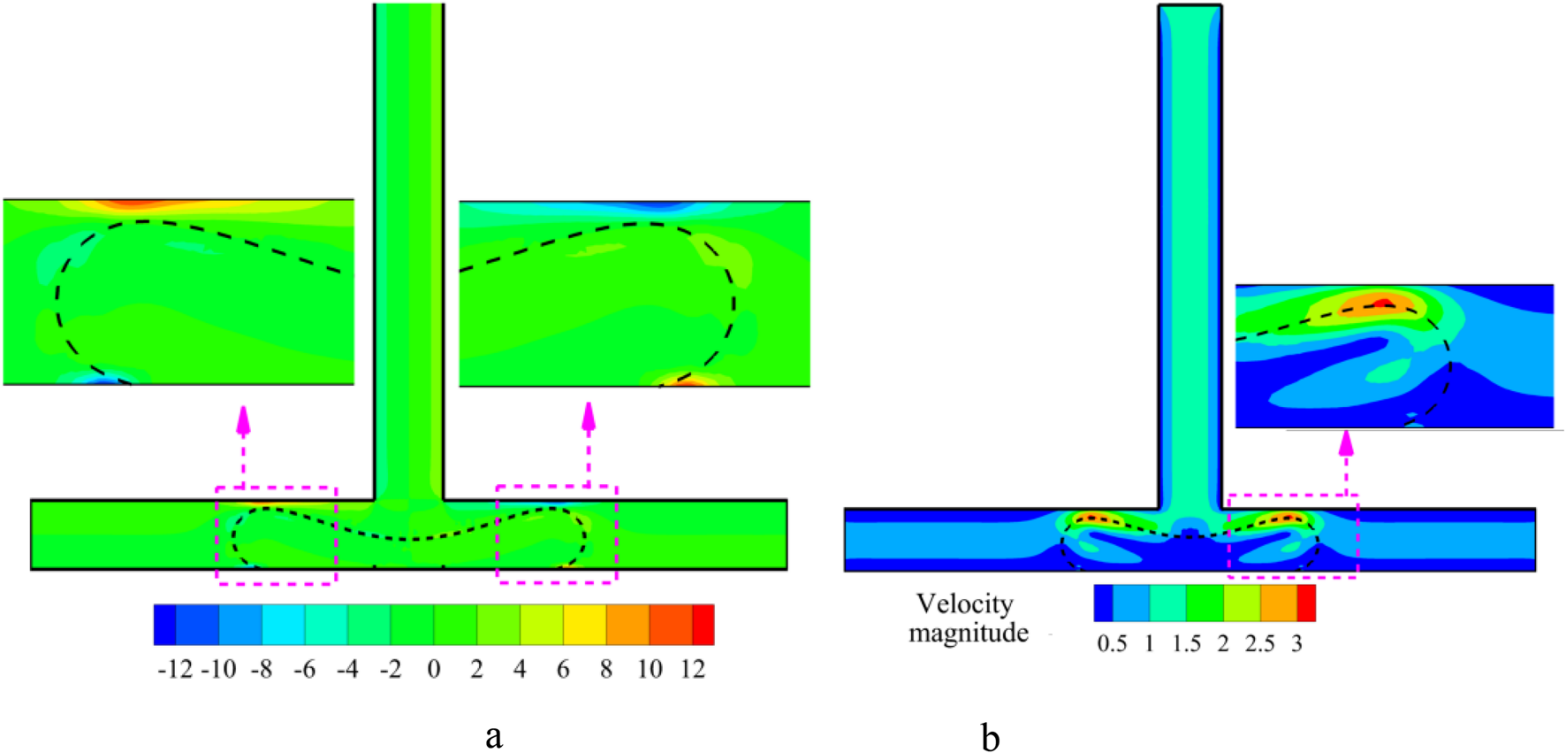
(**a**) Shear Stress Distribution and (**b**) Velocity Magnitude for Shape 4(a) at t* = 9.5.

The images in Figs. 5 and 6 show how the droplet moves in the symmetric T-junction microchannel for Ca = 0.013 at L* = 3.0 and L* = 3.5 with and without an obstacle for case I (B* = *b*/*W* = 1.0 and A* = *a*/*W* = 0.5) in the middle of the microchannel, respectively. The presence of the obstacle affects the flow pattern significantly. As can be seen in Fig. 5, the droplet pattern for L* = 3.0 shifts from non-break up pattern to break up one by the obstacle. As Fig. 6 illustrates, the flow pattern for L* = 3.5 is affected by the obstacle. Without the obstacle, the droplet breaks up with a tunnel at L* = 3.5, but with the obstacle, the flow breaks up without a tunnel at L* = 3.5. The droplet splitting depends on how strong the forces of pressure difference and shear are. When the pressure difference force is stronger, the continuous phase squeezes the back of the droplet until it splits into two smaller ones. This is called the breakup without tunnel regime. To better understand the effect of shear stress on the droplet breakup process, the shear stress distribution and velocity magnitude for Shape 5(a) at time t* = 9.5 are shown in Fig. 7a,b, respectively. As can be seen from the Fig. 7b, due to the formation of a tunnel, the velocity magnitude is also higher at the neck, indicating that this is the region of highest flow rate. The velocity gradient between the droplet surface and the wall increases, particularly at the neck. This elevated velocity gradient, in turn, results in a higher shear stress. Shear stress, as a force tangential to the surface, is directly proportional to the velocity gradient. The continuous phase flows between the droplet ends and the walls and rips the droplet apart and results in the shear force gets stronger and finally the mother droplet is divided in the daughter droplets.

**Figure 8:**
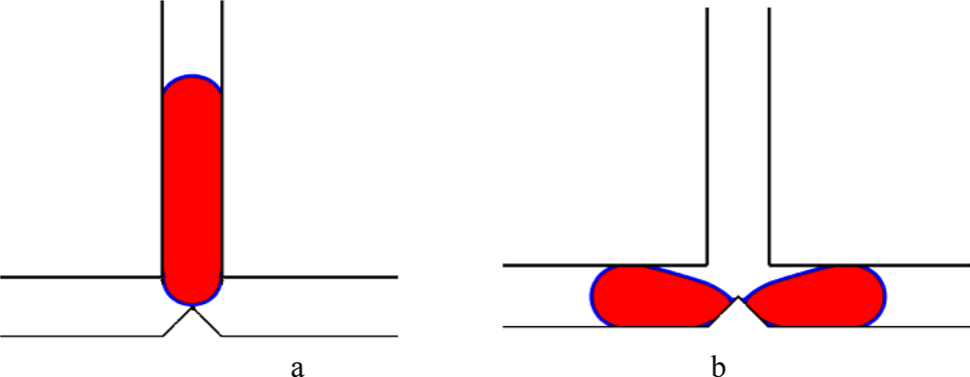
Two distinct moments during the droplet’s interaction with the triangular obstacle: (**a**) the moment when the droplet comes into contact with the tip of the triangle obstacle, and (**b**) the moment when the mother droplet undergoes breakup.

The breakup time (${\text{t}}_{breakup}^{*}$) is a key parameter that affects the droplet splitting process in microchannels. It is calculated by subtracting the time when the droplet reaches the edge of the triangle obstacle, $t_{i}$ (Fig. 8a), from the time when the droplet separates into two parts, $t_{b}$ (Fig. 8b). The formula is as follows:

6 $${\text{t}}_{breakup}^{*} = \frac{{(t_{b} - t_{i} ) U_{c} }}{W}.$$

**Figure 9:**
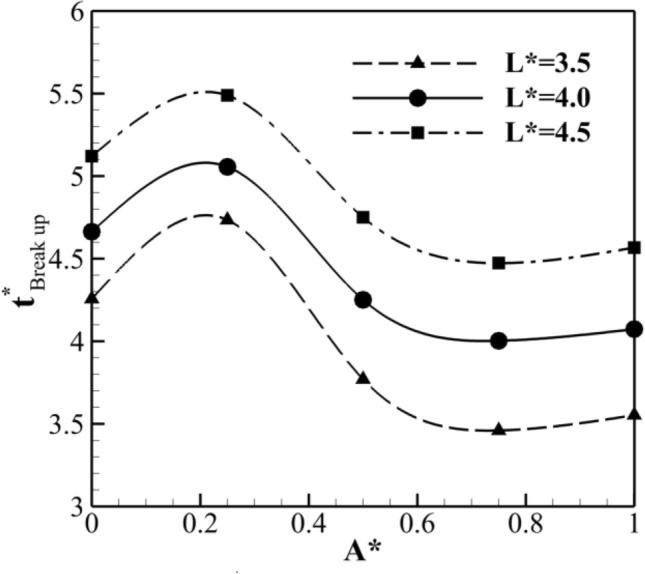
The non-dimensional time of breakup (${\text{t}}_{breakup}^{*}$) as a function of A* for various values of A* at Ca = 0.013 and B* = 1.0 for case I.

Figure 9 represents the non-dimensional time of breakup (${\text{t}}_{Break up}^{*}$) as a function of A* for various values of A* ranging from 0 to 1 at Ca = 0.013 and B* = 1.0 for case I. It is noteworthy that the shape of the droplet’s geometry plays a significant role in determining ${\text{t}}_{Break up}^{*}$. Specifically, A* = 0 indicates the absence of any obstruction or constraint at the center of microchannel, while A* = 1 signifies that the height of the triangular geometry is equal to the width of the channel. As evident from the figure, as A* increases from 0 to 0.25, the breakage time initially increases for all three values of L*. In this case, the droplet's movement encounters resistance at the center of microchannel, acting as a hindrance. As a result, the breakage time increases as A* increases from 0 to 0.25. However, as A* continues to increase further (0.25 to 0.75), ${\text{t}}_{Break up}^{*}$ starts to decrease. This suggests that beyond a certain point (approximately A* = 0.25), increasing A* leads to a reduction in ${\text{t}}_{Break up}^{*}$. When A* is between 0.25 and 0.75, the droplet's geometry includes a sharper tip and leads to a higher curvature at the tip region. The higher curvature can result in an increased Laplace pressure, which is the pressure difference across a curved interface. The elevated Laplace pressure can exert additional stress on the droplet, accelerating the breakage process. Interestingly, after reaching A* = 0.75, ${\text{t}}_{Break up}^{*}$ once again increases. This indicates that beyond this specific value of A*, the breakage time starts to increase again. It can be attributed to the obstructive nature of the droplet’s geometry and its impact on the flow dynamics. When A* exceeds 0.75, the droplet’s geometry includes a sharper tip that becomes more pronounced. This sharp tip acts as a hindrance to the droplet's movement through the channel. As the droplet tries to pass through a narrower region, it encounters increased resistance and difficulty in traversing the channel. Hence, the droplet faces more resistance and has a harder time undergoing the necessary deformation and breakup, resulting in a prolonged breakage process.

**Figure 10:**
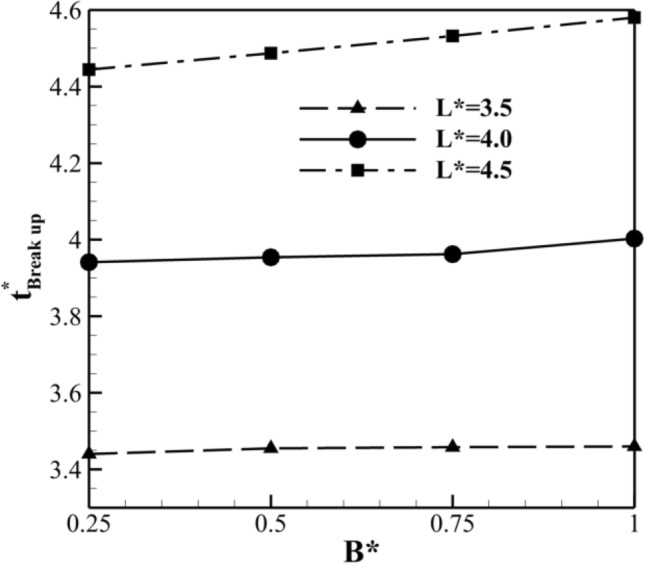
The non-dimensional time of breakup (${\text{t}}_{breakup}^{*}$) as a function of B* for various values of B* at Ca = 0.026 and A* = 0.75 for case I.

Based on Fig. 9, for A* = 0.75, the minimum value of ${\text{t}}_{Break up}^{*}$ occurs at different L* values. Therefore, the variation of ${\text{t}}_{Break up}^{*}$ at different B* values for A* = 0.75 at Ca = 0.013 and A* = 0.75 for case I are investigated and presented in Fig. 10. As evident, ${\text{t}}_{Break up}^{*}$ increases for different L* values, with increases of 0.5%, 1.5%, and 3% for L* = 3.5, 4.0, and 4.5, respectively. From Figs. 9 and 10, it is concluded that the minimum ${\text{t}}_{Break up}^{*}$ occurs at A* = 0.75 and B* = 0.25 for Ca = 0.013.

Utilizing asymmetric T-junctions provides the ability to split a mother droplet into arbitrary sizes, offering flexibility in droplet breakage. This knowledge has diverse applications and advantages. In microfluidics, it enables tailored chemical reactions, precise analysis, and improved device performance. In emulsions and cosmetics, it optimizes stability, appearance, and product quality. In biomedical research, it facilitates single-cell analysis, drug discovery, and advancements in genomics. In materials science, it allows for controlled synthesis of nanoparticles with desired properties. In inkjet printing, it ensures high-resolution printing and enhances graphics quality. Overall, the ability to control droplet breakup drives advancements across various fields, improving performance, product quality, and driving innovation. In the previous section, the breakage of the droplet under equal conditions (case I) was discussed. Now, the influence of the presence of an obstacle by halving the triangular geometry, transforming it into a right-angled triangle (case II) are investigated.

**Figure 11:**
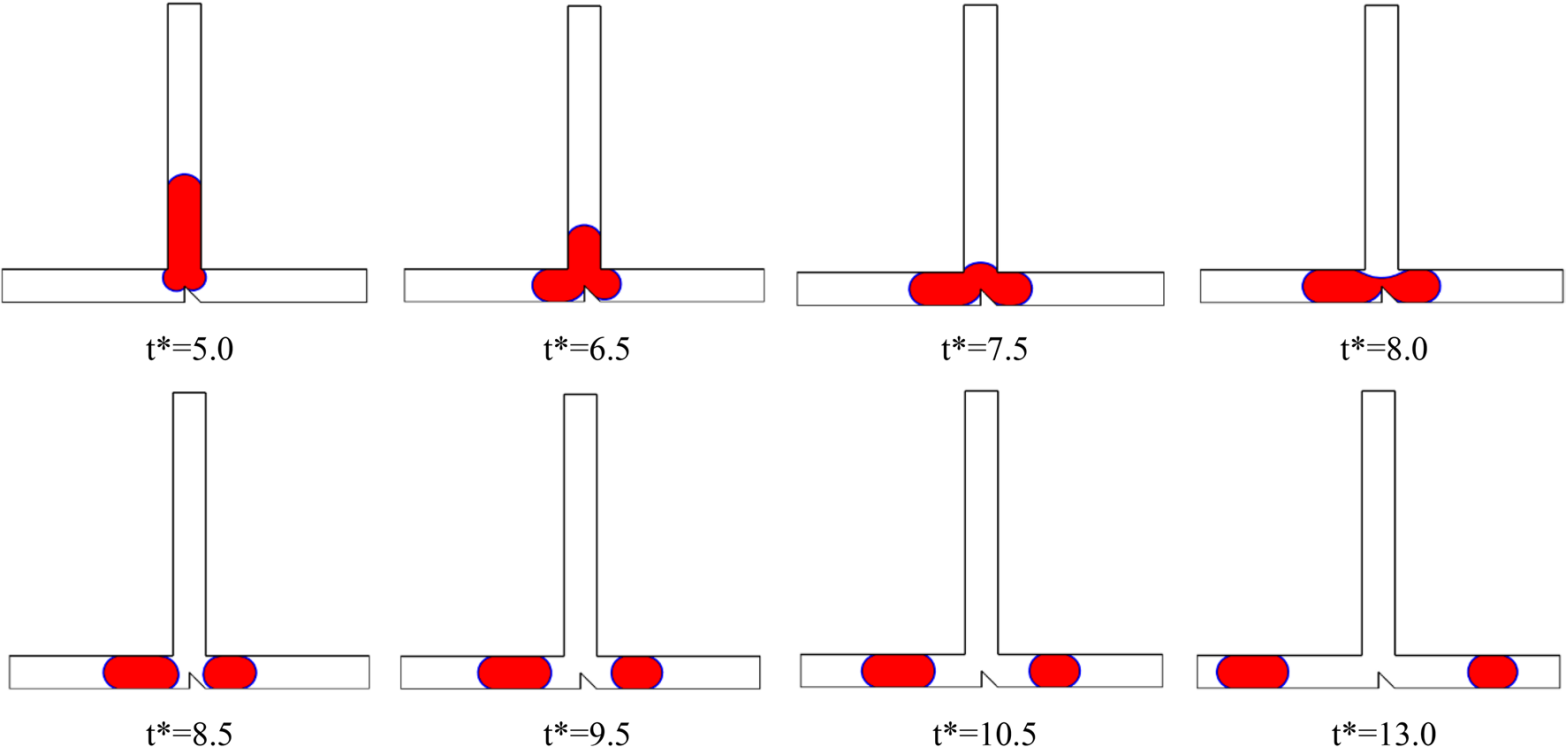
Snapshots of the droplet’s movement in the s T-junction microchannel at L* = 3.5 for Ca = 0.013 for case II (B* = *b*/*W* = 0.5 and A* = *a*/*W* = 0.5).

Figure 11 displays a series of snapshots illustrating the droplet motion in a T-junction microchannel with an obstacle for Case II (B* = *b*/*W* = 0.5 and A* = *a*/*W* = 0.5), where Ca is 0.013 at L* = 3.5. The images reveal an asymmetrical breakup process, resulting in the formation of two daughter droplets of unequal sizes. As the droplet's tip reaches the obstacle (t* = 6.5), it undergoes a transformation, developing two arms with independent menisci. The shape of the obstacle leads to a higher hydrodynamic resistance in the right arm compared to the left arm. Consequently, the larger meniscus penetrates into the left arm, while the smaller meniscus expands within the right arm (t* = 7.5). Subsequently, both menisci begin to flow in opposite directions (t* = 8), while the droplet's tip completely obstructs the continuous phase. Eventually, the mother droplet undergoes splitting, resulting in the formation of two daughter droplets with different sizes. Following the splitting event, both daughter droplets begin to move towards opposite sides of the microchannel, each flowing into one of the arms.

**Figure 12:**
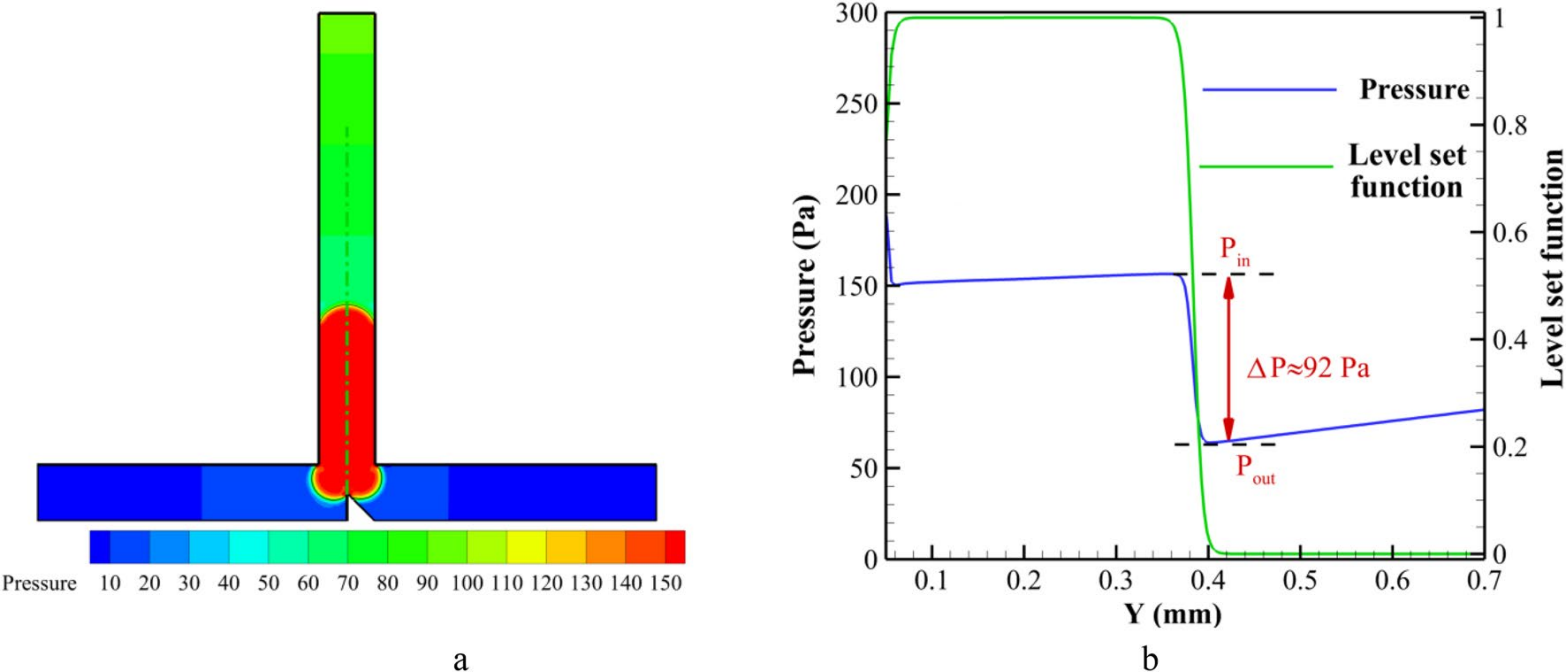
(**a**) Pressure field and (**b**) the pressure and Level set function distributions along the axis of the T-junction around the interface for t* = 5.0.

**Figure 13:**
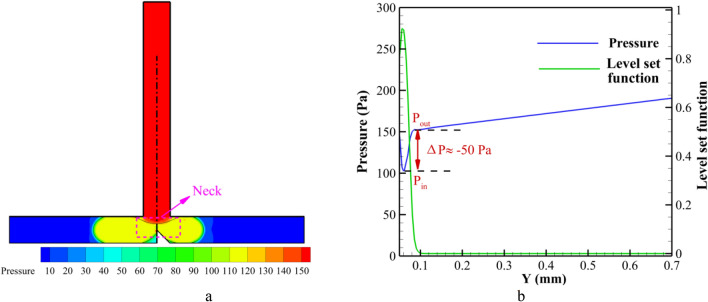
(**a**) Pressure field and (**b**) the pressure and Level set function distributions along the axis of the T-junction around the interface for t* = 8.0.

In an effort to gain a comprehensive understanding of the impact of pressure, Figs. 12 and 13 are presented to illustrate the pressure field, pressure distribution, and Laplace pressure drop across the rear droplet interface at specific time instances denoted as t* = 5 and 8, correspondingly. The depiction of the level set function in the figures serves the purpose of facilitating the identification of the rear interface of the droplet. Consequently, the pressure disparity across the said interface, referred to as the Laplace pressure $\Delta P$, can be quantified as the difference between the pressure inside ($P_{in}$) and outside ($P_{out}$ t) the droplet. When the curvature of the front part of the droplet comes into contact with the triangular region, there is a noticeable increment in the pressure within the droplet. Evidently, from the analysis of Fig. 12b, it is observed that the internal pressure and the pressure in the vicinity behind the droplet hover around the value of 92 pa. Upon examining Fig. 13 at t* = 8, a notable observation is made when the droplet completely exits the primary channel, resulting in the transformation of the interface into a concave shape, accompanied by the formation of a neck at the junction; this neck gradually elongates until the eventual occurrence of droplet breakup. The Laplace pressure ($\Delta P$) consistently maintains a negative magnitude of -50 pa, as visually portrayed in Fig. 13b.

**Figure 14:**
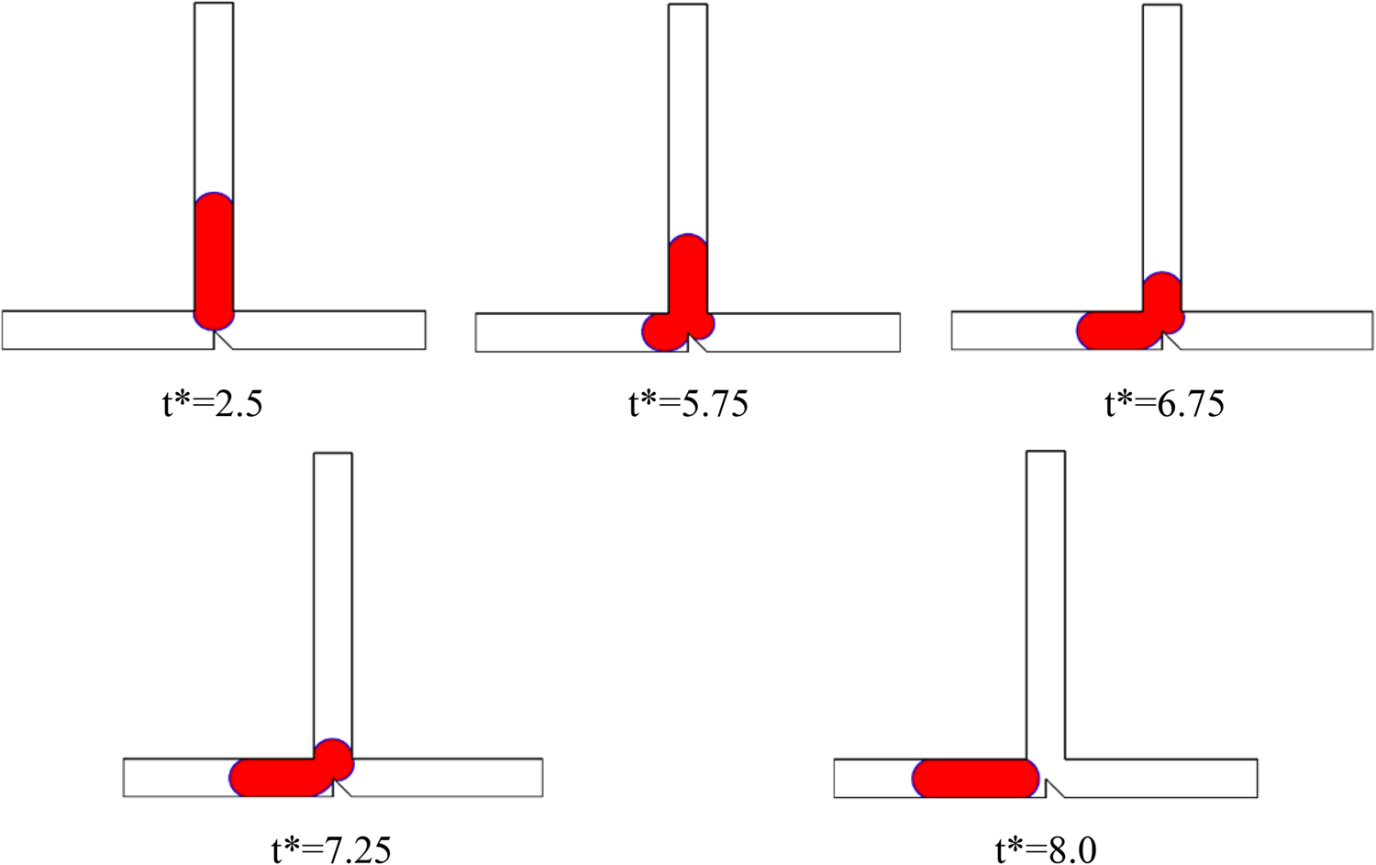
Snapshots of the droplet’s movement in the s T-junction microchannel at L* = 3.5 for Ca = 0.007 for case II (B* = *b*/*W* = 0.5 and A* = *a*/*W* = 0.5).

Figure 14 illustrates the progression of droplet motion in a T-junction microchannel with an obstacle for Case II (B* = *b*/*W* = 0.5 and A* = *a*/*W* = 0.5), where Ca is 0.007 at L* = 3.5. It is evident that the mother droplet remains unbroken, exhibiting a sorting pattern as it is entirely dragged towards the left branch of the microchannel. In comparison to Fig. 11, the capillary number has been decreased. When the capillary number is smaller, the surface tension force, which tends to minimize the surface area of the droplet, increases and becomes more dominant in comparison of shear stress and pressure difference forces. Hence, the droplet remains relatively intact and is guided towards the microchannel with lower resistance. Furthermore, there is a fundamental difference between the absence of droplet breakage in a symmetric T-junction microchannel without obstacles (Fig. 5a) and this configuration (Fig. 14). In which, the non-splitting droplet always moves to the left channel while that it randomly flows into either right or left of the outlet arm in the symmetric T-junction microchannel without obstacle.

**Figure 15:**
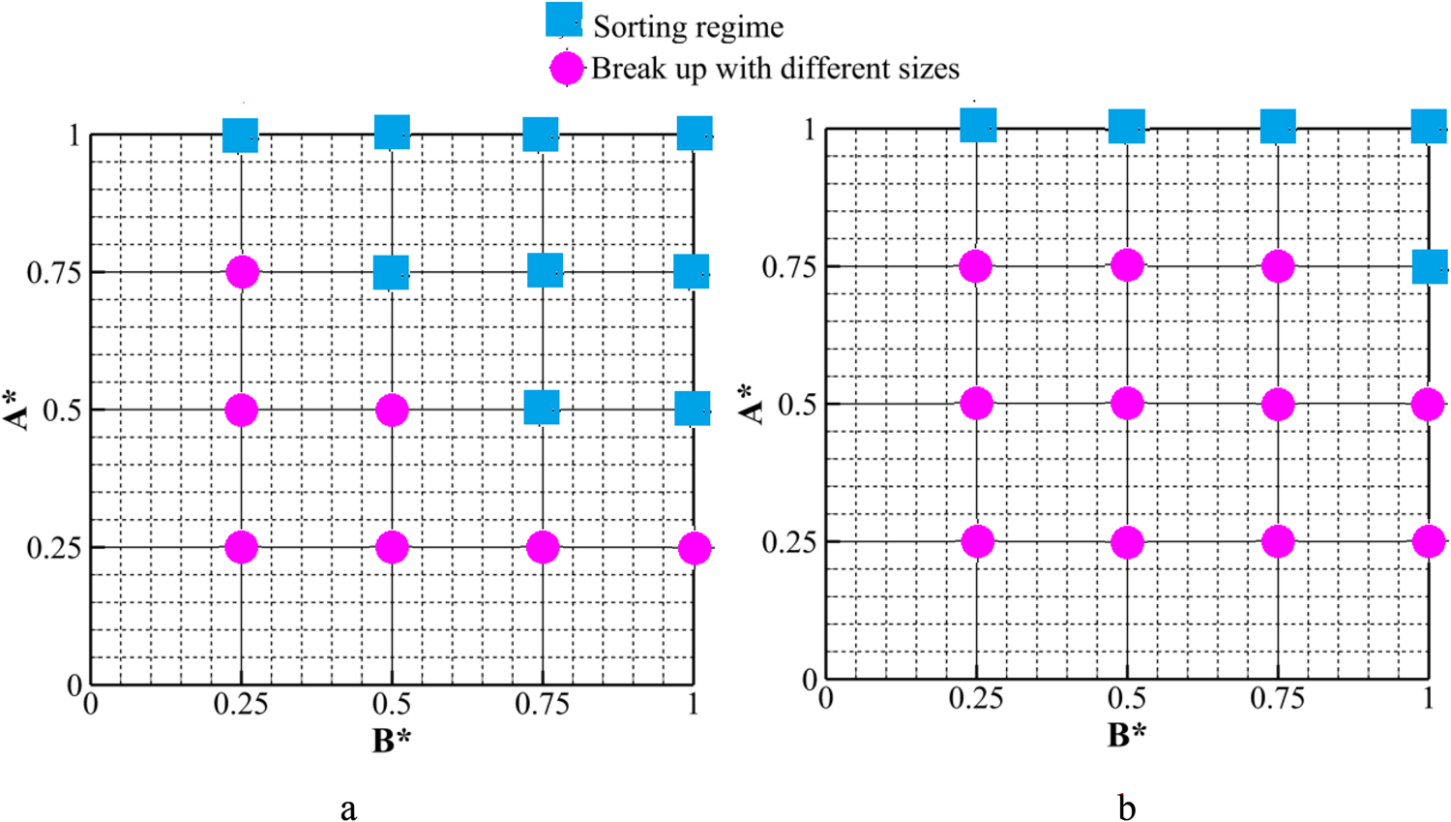
Phase diagram of A* versus B* for Case II at L* = 4.0: (**a**) Ca = 0.013 and (**b**) Ca = 0.026.

To gain a comprehensive understanding of the various states assumed by a droplet under different circumstances, the investigation takes into account a spectrum of values for A* and B* and subsequently consolidates the findings from the existing simulations into phase diagrams, as depicted in Fig. 15. For L* = 4, a series of 2D simulations are conducted with varying values of A* = 0.25, 0.5, 0.75, and 1.0, as well as B* = 0.25, 0.5, 0.75, and 1, within the range of Ca ∈ [0.013, and 0.026] for case II. It is evident that an elevated A* and B* result in the establishment of a sorting regime. Decreasing either A* or B* promotes the droplet breakup with different sizes. For A* = 0.25 and 0.5 at Ca = 0.026 and A* = 0.25 at Ca = 0.013, increasing the value of B* in the range of 0.25–1.0 only induces breakup regime. However, for A* = 0.5 and 0.75 at Ca = 0.013 and A*0.75, increasing B* in the range of 0.25–1.0, the regime changes from breakup regime to sorting regime. For A* = 1 at both Ca numbers, sorting regime is observed for all B*. It shows that when the height of triangle for case II is equal with the width of microchannel droplet does not break and it passes the left arm of microchannel. has a base length of *b* and a height of *a*.

Case II offers a significant advantage: it can effectively break a mother droplet into daughter droplets of various sizes. To fully exploit this capability, it is crucial to accurately predict the break-up ratio (BR*) of the droplets. BR* is a mathematical term defined as the ratio between the area of the right daughter droplet and the area of the left daughter droplet. This ratio typically falls between 0 and 1.

- BR* = 0: This indicates a scenario where the entire mother droplet goes into the left branch (sorting).
- BR* = 1: This represents a symmetrical breakup, where both daughter droplets have equal sizes.
- BR* = between 0 and 1: These represent breakups resulting in daughter droplets of different sizes.

**Figure 16:**
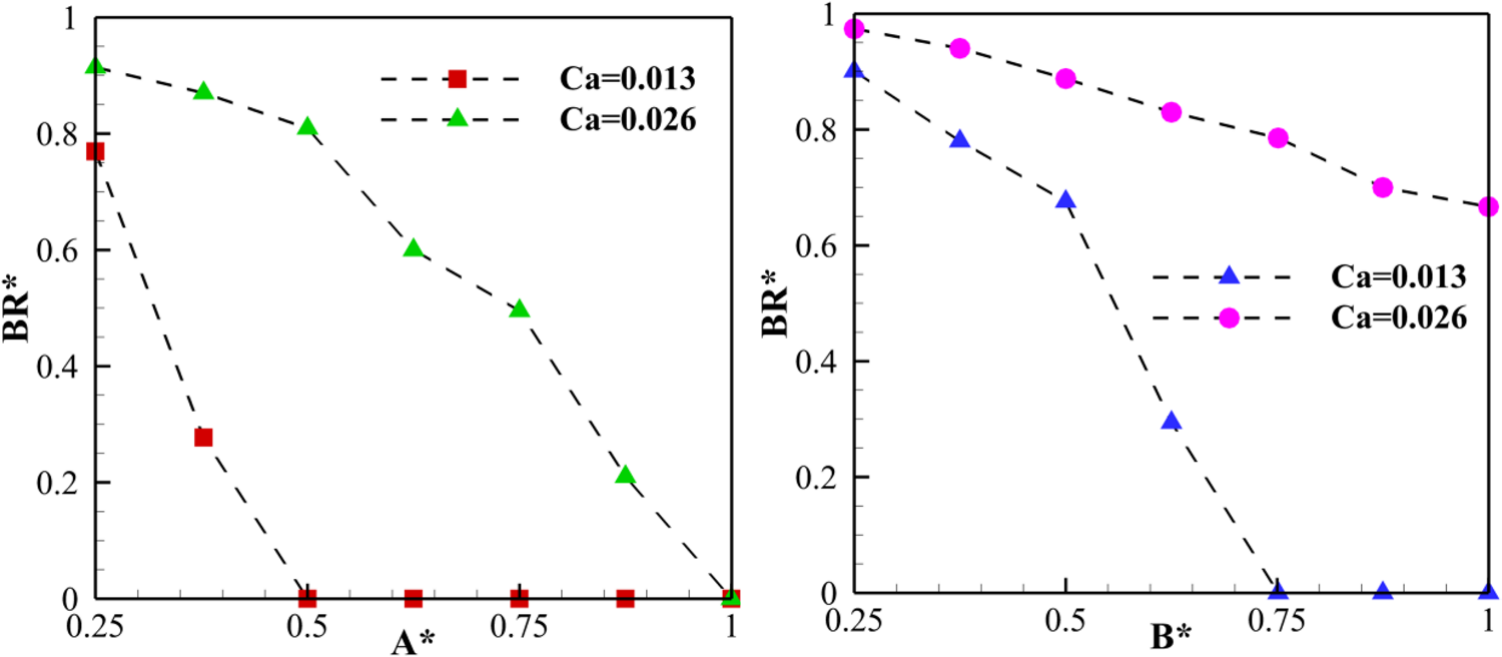
The break-up ratio of the droplets as a function of A* (left) and B* (right) at L* = 4.0 for Ca = 0.013 and Ca = 0.026.

Previous studies have shown that the breakup of a mother droplet in a channel without a triangular obstacle depends heavily on two dimensionless parameters: Dimensionless droplet length and Capillary number. However, in Case II, where a triangular obstacle is present, two additional dimensionless parameters (A* and B*) play a crucial role: Fig. 16 visually demonstrates how A* and B* influence BR* when L* is fixed at 4.0 and Ca has values of 0.013 and 0.026. The figure shows that BR* decreases as A* and B* increase, reaching a critical point. This means that as more volume of the mother droplet is drawn towards the left side of the channel (due to A* and B*), the resulting asymmetric breakup becomes more pronounced, with the left daughter droplet becoming larger. If A* and B* exceed a certain threshold, the entire mother droplet gets pulled towards the left branch, leading to a BR* of 0 (sorting). The figure also reveals that a higher Ca value (0.026) leads to a higher BR* compared to a lower Ca value (0.013) when A* and B* are constant. This suggests that stronger capillary forces (higher Ca) tend to promote more even breakups (higher BR*). Finally, the figure shows a sudden drop in BR* to 0 for lower values of A* and B* as the Ca value decreases. This suggests that weaker capillary forces (lower Ca) can lead to complete sorting (BR* = 0) even with relatively low values of A* and B*.

## Conclusion

In the current study, the effect of triangular obstacles on droplet breakup dynamics in microfluidic systems is simulated by COMSOL software. Two types of triangle obstructions, located at the bifurcation (case I) and aligned with the flow (case II), are considered to study their impact on droplet behavior. The influence of various parameters, including Capillary number, non-dimensional droplet length, non-dimensional height of the triangle, and non-dimensional base length of the triangle, is thoroughly examined. The results demonstrate that the mother droplet breaks up faster when the obstacle is positioned at the center of the microchannel. For the second studied case, the aim is to identify conditions where the droplet breaks up into unequal-sized droplets or remains intact under different flow conditions. The findings highlight five distinct patterns: no breakup, breakup without a tunnel, breakup with a tunnel, droplet breakup into unequal-sized droplets, and sorting. These patterns depend on the presence or absence of the triangular obstacle, type of it and the specific flow conditions. The results emphasize the significant influence of triangular obstacles on droplet breakup dynamics, with the obstacle shape and position playing crucial roles in determining the breakup characteristics.

## Data Availability

All data generated or analysed during this study are included in this published article.
